# Adherence Barriers to Antimicrobial Treatment Guidelines in Teaching Hospital, the Netherlands

**DOI:** 10.3201/eid1003.030292

**Published:** 2004-03

**Authors:** Peter G.M. Mol, Willem J.M.J. Rutten, Rijk O.B. Gans, John E. Degener, Flora M. Haaijer-Ruskamp

**Affiliations:** * University of Groningen, Groningen, the Netherlands; † University Hospital Groningen, Groningen, the Netherlands

**Keywords:** antibiotics, guidelines, implementation, prescribing-behavior, therapeutic drug use, qualitative research

## Abstract

To optimize appropriate antimicrobial use in a university hospital and identify barriers hampering implementation strategies, physicians were interviewed regarding their opinions on antimicrobial policies. Results indicated that effective strategies should include regular updates of guidelines that incorporate the views of relevant departments and focus on addressing senior staff and residents because residents do not make independent decisions in a teaching-hospital setting.

In an era of increasing bacterial resistance and the availability of a plethora of antimicrobial agents, hospitals have developed policies to promote prudent antimicrobial prescribing (*1*). The mainstay of such policies is preferably an evidence-based antimicrobial treatment guideline (*2*). Adherence to such hospital guidelines is often low to moderate (40%–60%) (*3*,*4*). Therefore, much effort is put into programs aimed at optimizing the antimicrobial prescribing practices of physicians. To plan an effective intervention strategy, however, one must know the extent to which clinicians perceive the need for a guideline and support implementing that specific guideline (*5*). The impact of different implementation strategies varies and when, and under what conditions, a particular strategy should be used is often not clear (*1*,*3*,*4*).

We examined barriers that existed in different groups of physicians to the use of a general, hospitalwide antimicrobial treatment guideline. A qualitative approach was chosen to maximize the identification of relevant issues, especially on content and development process of the guideline and physicians’ and organizational characteristics (*6*,*7*).

## The Study

Physicians were probed on their opinions on antimicrobial policies in general and on aspects of the current antimicrobial treatment guideline and its usefulness in daily clinical practice, using in-depth interviews lasting 20–45 minutes. That antimicrobial treatment guideline was drawn up by the hospitals’ antibiotic use committee, which was composed of specialists of relevant departments. Paper copies of the antimicrobial treatment guideline were distributed hospitalwide, in 1995, with an update in 1999. From the Department of Internal Medicine of the University Hospital, Groningen, physicians were recruited through their chief medical officers in October and November 2001. Interviewees were not paid; all involved were informed that interview-data would be strictly confidential to guarantee interviewees independence. One resident and one supervisor were interviewed from each of six internal medicine subspecialties—intensive care, general internal medicine, pulmonology, gastroenterology, nephrology, and hematology. Residents had 1–6 years of precertification training, and supervisors had been board-certified for 1 to 23 years as a specialist. From the group of infectious disease consultants, two clinical microbiologists and a consulting infectious disease specialist were interviewed. Each interview was concluded with a case-scenario to explore agreement between general opinions on antimicrobial use and response to a specific infectious disease case.

Interviews were audiotaped and transcribed verbatim; the content was analyzed by P.M. and W.R. One recording of an interview with a clinical microbiologist was damaged and could not be used. Recurrent topics were attributed to dominant themes. Important issues and themes emerging from previous interviews were incorporated into subsequent interviews. Themes were classified as barriers related to 1) the guideline, 2) physicians’ characteristics, and 3) characteristics of the institution. Interviewing and analysis were partly simultaneous, which is consistent with the grounded theory approach (*8*). Physicians were interviewed once. After 15 physicians had been interviewed, no new issues came up, and we stopped interviewing.

### Barriers Related to the Guideline

All physicians but one were aware of the guideline, although six never had received a personal copy (Table 1). They suggested that more effort should be put into familiarizing physicians with the guideline. Residents preferred an electronically available copy of the guideline. All physicians agreed with the basic principle of the guideline: an initially empirical antimicrobial treatment should be streamlined to the most narrow-spectrum antimicrobial agent effective against isolated pathogens. Physicians stressed that the guideline needed to be consistent with existing policies, concise, and up-to-date. Supervisors’ expected their own prescribing to be consistent with the guideline, without actually knowing its contents, though residents experienced the opposite: residents experienced that supervisors regularly prescribed or advised them to prescribe antibiotics that were not recommended by the hospital guideline. Infectious disease consultants, as members of the antibiotic use committee, had contradictory views on one aspect of the contents of the guideline. They supported its recommendations for using aminoglycosides when appropriate but were reluctant to advise prescribing them for individual patients.

**Table 1:** Quotations illustrating themes

Level of	Quotation	
Guideline characteristics	“It would be great to have an electronic version” or “…even better everyone a handheld PDA with guideline.” [R1]^a^ “You have to go with the flow and not necessarily against it…otherwise you will have a hard time |getting the guideline accepted|.^b^” [S1]	
Physicians’ characteristics	“I often look in the booklet |guideline| especially for those indications that you do not encounter much…” [R4] “…you sometimes get these incomplete results: just a gram-stain without sensitivity results, I then rather wait for the complete results” [R5] “…they |residents| do not look at the quality of the |culture and sensitivity| tests” [ID] “I would continue. |with broad-spectrum therapy even when a cultured pathogen is sensitive to a more narrow spectrum agent| The patient is doing well, he is responding to his antibiotic therapy. I would not streamline at this time. I do not see any reason. ...Never change a winning team.” [R1] “My autonomy… as a lung specialist I feel I have and can decide on |what treatment for| pulmonary infections” [S2] “…for the large majority of patients it |choice of antibiotic| is just very clear, it is just a formality.” [S3] “In our department? I cannot remember the time we had resistant pneumococci. We know they exist and we remind each other of that, but we have never had that here.” [S2] “..horrible..I can imagine obliging people to use it |registration form| in the framework of their training… but to have to defend this as a standard measure, I do think this goes too far.” [S5] “A wonderful idea… it could be quite a guiding instrument ... it should be education and intervention.” [ID]	
Social and institutional context	“…and when you move to another department you learn within a week their |supervising specialists| prescribing preferences… they will just keep an eye on you.” [R4] “I would first consult my supervisor and if he gets stuck I would ring up a microbiologist.” [R5]	

^a^R, resident; S, supervisor; ID, infectious disease specialist. ^b^|Text| is added by the author for clarification. This additional text is merely meant to clarify a physician’s statement, and we have made every effort to not altering the implication of any statement

### Barriers Related to Physicians’ Characteristics

Residents were more receptive to using the guideline than were supervisors, especially for rare infectious diseases because they lack experience and have to look up the most effective therapy for a specific condition more often. Junior residents acknowledged a lack of knowledge in interpreting culture and antimicrobial sensitivity test results, resulting in problems with effectively using the guideline based on such tests (Table 1). Infectious disease consultants shared this concern. In contrast to their statements supporting streamlining antimicrobial therapy, residents reported that they were not inclined to change therapy with an effective broad-spectrum antimicrobial agent, once the pathogens’ sensitivity test results became available.

Supervisors did not perceive a strengthened antibiotic policy as an advantage because they considered guidelines a threat to their professional autonomy and as interfering with daily clinical practice. Prescribing an antimicrobial agent was often considered a routine activity. Supervisors doubted the need for an antimicrobial use policy, which was reinforced by the fact that they did not perceive many problems with antimicrobial resistance in daily clinical practice.

At the time of the interviews, a paper “critical-pathway”^1^ was discussed as a possible decision support tool for improved antimicrobial therapy. Supervisors and residents were negative towards such a tool. Supervisors considered it an unnecessary and unacceptable infringement of daily clinical work, while residents were mostly concerned about the added paperwork. The infectious disease consultants had great trust in a “critical-pathway” to guide antimicrobial drug prescribing, welcoming its educational value and potential for improving actual prescribing behavior.

### Social and Institutional Context

Residents in most teaching hospitals are not independent decision makers, and experienced specialists supervise their prescribing choices (Table 1). Residents run the day-to-day clinical care of patients in our hospital; they rotate to different departments at 4-month intervals and have to adapt each time to the mores of a new department or supervisor. They considered the antimicrobial-treatment guideline a helpful tool in coping with existing differences between departments; some departments had their own protocols but mostly discussed antimicrobial use policies informally in departmental patient reviews. The role of the infectious disease consultant was one of adviser. Residents would primarily seek advice from their supervisor, and the final decision is always made by the supervisor.

### Case Scenario

To further ascertain the physicians’ use of the antibiotic treatment guideline, we presented a scenario for a case of community-acquired pneumonia (Appendix). All physicians, except for one supervisor, began the patient’s treatment with broad-spectrum antimicrobial agents. Residents were hesitant to streamline initial therapy, fearing that such changed therapy might be clinically less effective. Infectious disease consultants and supervisors streamlined therapy based on gram-stain results only.

## Conclusion

Our findings support earlier study findings that an intensive implementation strategy is needed for physicians to make their prescribing practices consistent with guideline recommendations. Table 2 shows the identified barriers along with our suggestions about which interventions might be effective. Any implementation process passes through different stages, each requiring a different intervention approach (*9*). The supervisors are in an early stage of such a process; they need to be motivated to use the antimicrobial-treatment guideline and to change their prescribing behavior accordingly. Clear involvement in the development of the antimicrobial-treatment guideline may overcome reservations of supervisors with regard to feelings of losing their autonomy. Supervisors see no need to follow the guideline recommendations; they do not perceive antimicrobial resistance as a problem, which may be understandable in view of the low resistance patterns in Dutch hospitals (*10*). Their routine decision-making leaves little room for guideline consultations. Providing feedback on their own and departmental prescribing patterns may identify areas to be improved and raise awareness of a need to change (*11*,*12*). The usefulness of the guidelines could be emphasized for nonroutine cases, about which physicians were less reluctant to consult the guideline.

**Table 2:** Barriers and proposed interventions

	Barriers identified	Proposed interventions
Guideline	1. Dissemination 2. Credibility of content	1. Develop and actively distribute hard-copy and electronic version 2. Incorporate departmental policies, and update regularly − For both 1 and 2, organize meetings to introduce guidelines and set up an active outreach committee
Physician *Readiness to change or use the guideline*	*Supervising specialists* 3. No need for a guideline, because − Routine prescribing − No perceived resistance problems 4. Autonomy	3. A combination of group and individual feedback (“academic detailing”) to supervisors and residents 4. Incorporate specialists/departmental views in guideline (see 2, above)
	*Residents* 5. Insufficient knowledge − Of culture results − Low self-efficacy regarding streamlining	5. Active educational support on interpretation of culture-results and for streamlining therapy
	*Infectious disease consultants* 6. Overestimate the feasibility of an intervention	6. Check support before implementation of an intervention
Social and institutional context	7. Residents are not independent decision makers and their prescribing decisions are supervised by specialists 8. Infectious disease consultant secondary to supervisor 9. Different guidelines between departments	7. Target both residents and supervising specialists 8. Target supervisor, formalize advice of consultants 9. Incorporate departmental policies (see 2 and 5, above)

Residents are more open to using the guidelines; they are willing to adopt the recommendations because it helps them in their learning process, making them ideal candidates for interventions. For them, the barrier to be addressed is whether streamlining is safe. One way of affirming this is facilitating a better understanding of culture and sensitivity tests, for example, through infectious disease consultants’ support (*6*). As paper critical-pathways will not suffice, face-to-face educational visits, so-called academic detailing, may be a better way to improve residents’ prescribing practices (*13*). Academic detailing should focus not only on interpretation of test results but also on acting on the implications. Infectious disease consultants should be motivated to give advice consistent with the guideline.

In an institutional context where residents are not independent decision makers, any implementation plan should combine strategies aimed at both residents and supervisors. For residents who change departments regularly, a generally adopted hospitalwide guideline facilitates a consistent learning environment and increase their rational decision making. Addressing the role model function of supervisors for residents may be one more way to motivate them to use the guideline, in view of the impact that supervisors have on residents (*14*).

The limited number of physicians interviewed in this study is in line with a qualitative research approach aimed at generating hypotheses (*15*). We found physicians to be very open in expressing their, sometimes negative, views during the interview sessions. Residents were quite frank about their relationship with their supervisors, possibly because the interviewer had no direct link to any chief medical officer and confidentiality was assured.

In conclusion, intervention strategies should focus on improving dissemination and credibility of the recommendations, focusing on both supervisors and residents, although each group needs a tailored approach. Active outreach, as in face-to-face educational visits, may be the best approach to tackling the various barriers in one intervention program aimed at optimizing antimicrobial use.

## Footnotes

1 A paper “critical-pathway” combines an antimicrobial drug order form with a decision support tool. Filling out a few relevant case-characteristics guides the prescriber to the guideline’s recommendation for that specific case.

## Appendix: Case Scenario

The following scenario was presented for a case of community-acquired pneumonia.

A male patient aged 63 years, with hypertension treated with metoprolol and hydrochlorothiazide, is referred by a local general practitioner to the emergency department of your hospital at 11:30 p.m. He has a temperature of 40°C and is dyspneic but not confused. Physical examination reveals only crackles and egophony in the right lower lung field.

Question (Q) 1. What additional examinations would you request?

Q2. What kind of therapy would you suggest?

The next morning a chest x-ray shows infiltration in the right lower lung field, and the sputum culture shows gram-positive diplococci.

Q3. Does this influence your therapeutic decision?

Two days later the patient is improving and the fever has subsided. Blood-culture results read literally as follows: *Streptococcus pneumoniae*, sensitive to penicillin G, amoxicillin, amoxicillin with clavulanic acid and cefuroxim.

Q4. Do you adapt your therapeutic choice?

Hospital Guideline on Community-Acquired Pneumonia

For this straightforward case of a patient with a clearly community-acquired pneumonia, the initial empirical treatment according to the hospital guideline would be amoxicillin/clavulanic or cefuroxim. The guideline recommends streamlining antimicrobial therapy to intravenous penicillin G or oral amoxicillin based on sensitivity-tests of the isolated *S. pneumoniae.*
